# Hidden in Plain Sight: A Case Series of Inflammatory Bowel Disease With Dermatologic Lesions As Initial or Concurrent Manifestations

**DOI:** 10.7759/cureus.55548

**Published:** 2024-03-05

**Authors:** Kinan Obeidat, Hamza Salim, Jordan C Malone, Hwe Won Lee, Sheharyar Merwat

**Affiliations:** 1 Department of Internal Medicine, University of Texas Medical Branch at Galveston, Galveston, USA; 2 Department of Gastroenterology and Hepatology, University of Texas Medical Branch at Galveston, Galveston, USA

**Keywords:** erythema nodosum, bloody diarrhea, crohn’s disease (cd), ulcerative colitis (uc), inflammatory bowel disease, pyoderma gangenosum

## Abstract

Pyoderma gangrenosum (PG) and erythema nodosum (EN) are rare skin conditions associated with inflammatory bowel disease (IBD), with increasing incidence as the disease progresses. We describe three cases of newly diagnosed IBD with cutaneous extraintestinal manifestations (EIMs) at the time of diagnosis. Three previously healthy patients presented with bloody diarrhea and concomitant nodular and ulcerating skin lesions at the onset of diarrhea. Dermatopathology showed PG and EN with endoscopic confirmation of ulcerative colitis. Clinical improvement was achieved with steroids and biological agents. These cases display the importance of a proper review of symptoms and a detailed workup of dermatological lesions prior to assuming infectious etiology.

## Introduction

Erythema nodosum (EN) and pyoderma gangrenosum (PG) are cutaneous conditions with an incidence of only 1-5 cases per 100,000 persons and 3-10 cases per million people, respectively [1,2]. EN, an acute nodular septal panniculitis, and PG, a rare ulcerative, noninfectious neutrophilic dermatosis, are commonly associated with inflammatory bowel disease (IBD). EN is the most encountered cutaneous extraintestinal manifestation (EIM) of IBD, occurring in up to 3% of patients with ulcerative colitis (UC) and up to 8% of patients with Crohn's disease (CD) [3-5]. PG is the second most common cutaneous EIM seen in IBD, occurring in only 0.5%-5% of patients [6]. We present three patients, with no significant gastrointestinal past medical history, who presented to our institution with worsening cutaneous lesions and new-onset hematochezia.  

## Case presentation

Case 1  

**Figure 1:**
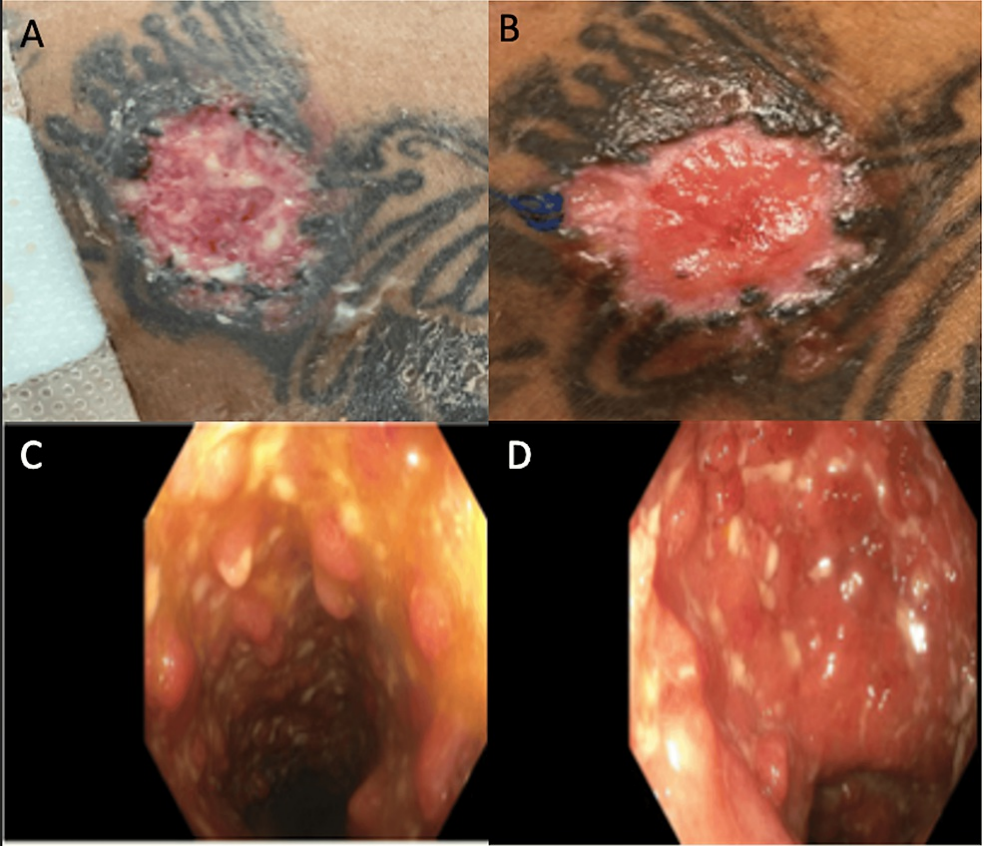
Cutaneous and endoscopic image findings A: Right shoulder lesion on initial presentation; B: shoulder lesion after three days of steroid therapy; C-D: pancolitis seen on colonoscopy

A 36-year-old healthy male presented with several weeks of worsening profuse bloody diarrhea, associated with abdominal pain, weight loss, and malaise. He reported a worsening right anterior shoulder lesion that was present prior to his gastrointestinal complaints and was refractory to treatment with antibiotics. Similar lesions were present on his left thigh and shin that were progressing. Lab evaluation was significant for leukocytosis and elevated inflammatory markers. C-reactive protein (CRP) was measured at 27.4 mg/dL, and erythrocyte sedimentation rate (ESR) was measured at 65 mm/HR. Imaging revealed pancolitis with perirectal and right lower quadrant reactive lymphadenopathy. Dermatopathology of the lesions revealed diffuse dermal neutrophilic infiltrates, consistent with PG. Colonoscopy revealed inflammation characterized by congestion, erosions, erythema, loss of vascularity, mucus, and pseudopolyps in a continuous and circumferential pattern from the rectum to the cecum with no colonic sites spared (see Figure 1). Colonic tissue samples returned positive for chronic active colitis with moderate lymphoplasmacytosis and crypt architectural distortion consistent with ulcerative pancolitis. He was started on steroids with a marked improvement in abdominal pain, hematochezia, and the appearance of skin lesions. He initiated adalimumab as an outpatient with continued improvement. 

Case 2  

**Figure 2:**
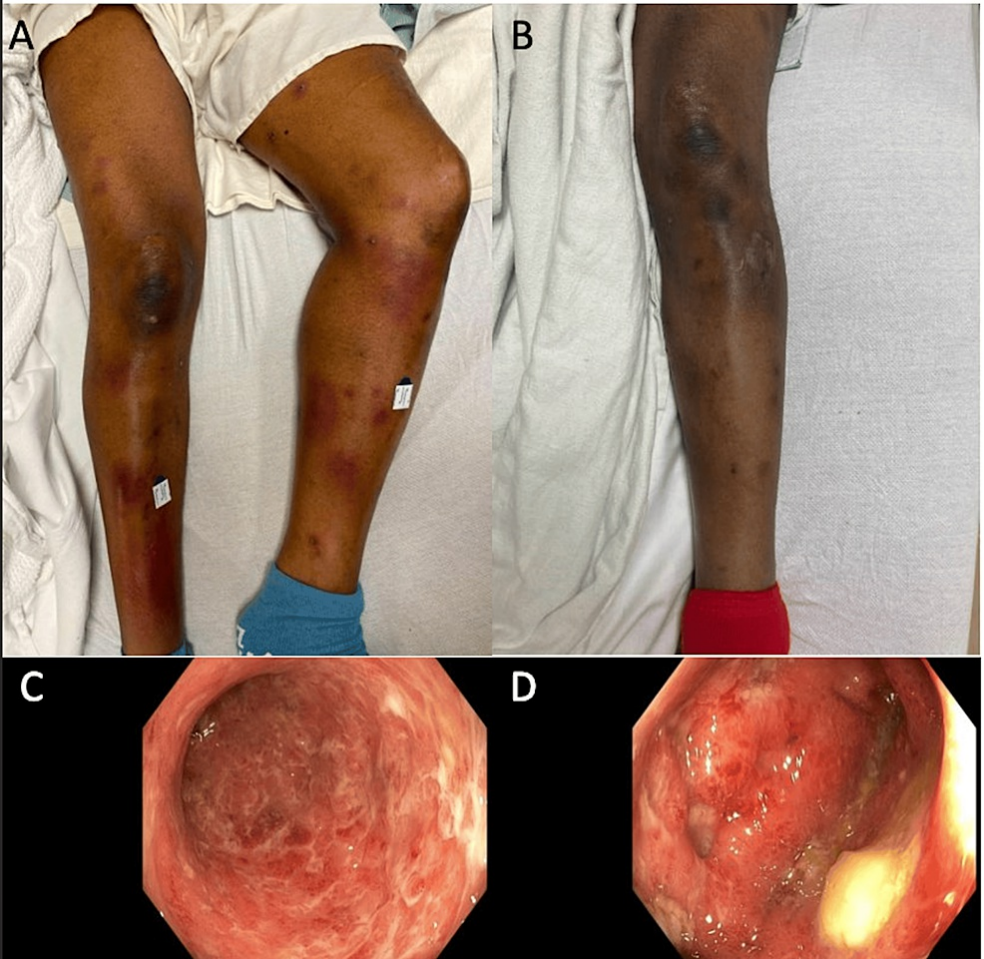
Cutaneous and endoscopic image findings A: Lower extremity cutaneous appearance on admission; B: left lower extremity one week after therapy with marked improvement; C: rectum on colonoscopy showing circumferential edema; D: sigmoid colonic edema seen during colonoscopy

A 48-year-old healthy male presented with worsening hematochezia and a painful, diffuse rash for one month prior to his presentation. Physical exam revealed a tender maculopapular rash and pustules on the torso, occiput, scrotum, and bilateral thighs. Infectious workup, including cytomegalovrius, returned negative, but laboratory evaluation showed significant leukocytosis. Additionally, inflammatory markers returned elevated with CRP at 32.4 mg/dL and ESR at 77 mm/HR. Imaging revealed marked thickening with mucosal enhancement up to the distal transverse colon consistent with proctocolitis. Dermatopathology of the lesions revealed septal panniculitis with focal granulomas and neutrophilic inflammation consistent with EN. Colonoscopy revealed inflammation characterized by edema, erosions, erythema, loss of vascularity, and mucus in a continuous and circumferential pattern from the rectum to the cecum with no colonic sites spared (see Figure 2). Pathology revealed moderate chronic active inflammation with multifocal active cryptitis, crypt abscesses, extensive crypt architectural distortion, and lymphoplasmacytosis consistent with ulcerative pancolitis. His skin lesions and symptoms markedly improved with steroid therapy. Adalimumab was initiated as an outpatient with continued improvement. 

Case 3 

**Figure 3:**
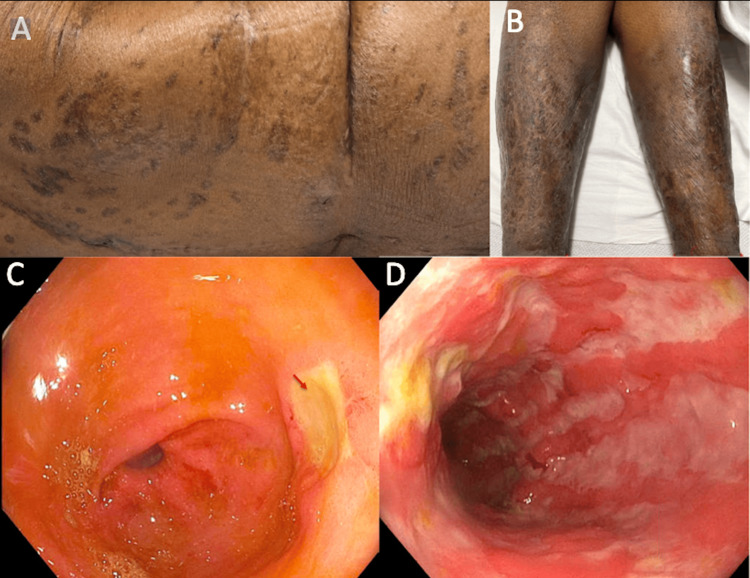
Cutaneous and endoscopic image findings A: Back cutaneous findings with dermatitis and scarring; B: skin findings on lower legs also with dermatitis and scarring; C: nonbleeding cratered ulcers in gastric antrum found during EGD; D: severe, diffusely erythematous friable, granular, and ulcerated sigmoid mucosa on colonoscopy

A 39-year-old female with history of cystic acne and an unspecified autoimmune skin disorder presented with large volume hematochezia in the setting of three months of weight loss, lower abdominal pain, and lower extremity edema. She noted a 20-year history of painless large clean-based ulcers on her legs and back which would resolve temporarily with topical steroids. An extensive prior workup yielded no diagnosis. Initially presented with hypotensive shock requiring massive transfusion and elevated inflammatory markers. Physical exam revealed multiple nonulcerated, nonerythematous scars on her back and legs. Imaging showed diffuse abdominal wall edema, bilateral sacroiliitis, and a featureless colon concerning for IBD without active inflammation. Dermatopathology of the lesions showed pigment incontinence, consistent with post-inflammatory hyperpigmentation and concerning for PG. Esophagogastroduodenoscopy revealed patchy, moderately erythematous mucosa without bleeding in the gastric body as well as multiple nonbleeding, cratered gastric, and duodenal ulcers. Flexible sigmoidoscopy showed erythematous, friable, granular, and ulcerated mucosa in the rectum, sigmoid colon and descending colon, with a few ulcers in the distal rectum (see Figure 3). Pathology showed crypt abscesses with crypt branching and distortion and increased plasma-lymphocytic inflammation consistent with ulcerative colitis. The patient responded to mesalamine and steroids, eventually switching to infliximab as an outpatient with continued symptomatic improvement. 

## Discussion

IBD is characterized by inflammation of the intestinal mucosa often leading to abdominal pain, diarrhea, weight loss, and rectal bleeding. In addition to gastrointestinal symptoms, the prevalence of EIM in patients with IBD ranges from 6% to 47% [7]. It has been reported that nearly 50% of IBD patients will suffer from at least one EIM at 30 years postdiagnosis with peak presentation at 90 months following diagnosis [7]. Dermatological lesions are established associations of IBD, with EN noted more frequently than PG in UC and Crohn’s disease [8]. These cutaneous lesions are often misdiagnosed and mistreated, especially if they are present prior to an established diagnosis of systemic illness such as IBD.

In clinical practice, we uncommonly see the classic presentation of newly diagnosed IBD with textbook dermatological manifestations such as PG or EN. Similar cases have been described that presented without classic gastrointestinal symptoms and instead presented with nonspecific complaints of malaise or weight loss in addition to progressive cutaneous lesions, which subsequently led to a delay in proper diagnosis of underlying IBD [9]. In our described cases, dermatologic lesions were either present prior to or concurrent with intestinal manifestations and were the most concerning pathology to our patients. Furthermore, these cases exhibit how EIMs of IBD can be commonly misdiagnosed, especially if they present early in the course of IBD and prior to other systemic symptoms which can lead to negative consequences. One of our patients had dermatologic lesions without a specific diagnosis for 25 years before gastrointestinal manifestations. In a similar case, PG was misdiagnosed as a resistant infectious lesion and subsequently managed with repeated surgical debridement resulting in worsening skin lesions due to the phenomena of pathergy [10]. Treatment of EN and PG should consist of treating the underlying cause. Corticosteroids and biological therapy, especially infliximab, have proven to be the most effective [11].

## Conclusions

These cases reinforce the importance of an accurate history, review of symptoms, and physical exam. Additionally, accurate workup of dermatological lesions is crucial prior to assuming infectious etiology. These necessary skills will undoubtedly steer the clinician toward a diagnosis of an underlying inflammatory process such as IBD. 

## References

[1] Schwartz RA, Nervi SJ (2007). Erythema nodosum: a sign of systemic disease. Am Fam Physician.

[2] Monari P, Moro R, Motolese A (2018). Epidemiology of pyoderma gangrenosum: results from an Italian prospective multicentre study. Int Wound J.

[3] Farhi D, Cosnes J, Zizi N (2008). Significance of erythema nodosum and pyoderma gangrenosum in inflammatory bowel diseases: a cohort study of 2402 patients. Medicine (Baltimore).

[4] Karmiris K, Avgerinos A, Tavernaraki A (2016). Prevalence and characteristics of extra-intestinal manifestations in a large cohort of Greek patients with inflammatory bowel disease. J Crohns Colitis.

[5] Lakatos L, Pandur T, David G, Balogh Z, Kuronya P, Tollas A, Lakatos PL (2003). Association of extraintestinal manifestations of inflammatory bowel disease in a province of western Hungary with disease phenotype: results of a 25-year follow-up study. World J Gastroenterol.

[6] Weizman AV, Huang B, Targan S (2015). Pyoderma gangrenosum among patients with inflammatory bowel disease: a descriptive cohort study. J Cutan Med Surg.

[7] Vavricka SR, Schoepfer A, Scharl M, Lakatos PL, Navarini A, Rogler G (2015). Extraintestinal manifestations of inflammatory bowel disease. Inflamm Bowel Dis.

[8] Vavricka SR, Rogler G, Gantenbein C (2015). Chronological order of appearance of extraintestinal manifestations relative to the time of IBD diagnosis in the Swiss inflammatory bowel disease cohort. Inflamm Bowel Dis.

[9] Shahid S, Myszor M, De Silva A (2014). Pyoderma gangrenosum as a first presentation of inflammatory bowel disease. BMJ Case Rep.

[10] Ye MJ, Ye JM, Wu L, Keating CP, Choi WT (2014). A challenging diagnosis: case report of extensive pyoderma gangrenosum at multiple sites. Clin Cosmet Investig Dermatol.

[11] Tavarela Veloso F (2004). Skin complications associated with inflammatory bowel disease. Aliment Pharmacol Ther.

